# Prospective Associations of Maternal Depressive Symptoms and Emotion Dysregulation with Children’s Internalizing Problems: The Moderating Role of Fathers

**DOI:** 10.21203/rs.3.rs-4209113/v1

**Published:** 2024-04-09

**Authors:** Yihui Gong, Xin Feng, Meingold Hiu-ming Chan, Natasha Slesnick

**Affiliations:** The Ohio State University; The Ohio State University; University of British Columbia; The Ohio State University

**Keywords:** Parental depression, Parental emotion dysregulation, Internalizing, Preschoolers

## Abstract

The occurrence of internalizing symptoms is prevalent among young children and can be observed as early as preschool years. Using a longitudinal approach, this study examined the moderating role of paternal depressive symptoms/emotion dysregulation in the prospective associations between maternal depressive symptoms/emotion dysregulation and children’s internalizing problems (depressive and anxiety symptoms). Ninety-four preschoolers and their mothers and fathers participated in the study. Mothers and fathers completed online questionnaires for all variables when their children were 4 years old and one year later. The results indicated that paternal depressive symptoms moderated the association between maternal emotion dysregulation and children’s later depressive, but not anxiety, symptoms. Specifically, higher levels of depressive symptoms in fathers exacerbated the negative influence of maternal emotion dysregulation on children’s later depressive symptoms, whereas fathers with low levels of depressive symptoms served a protective role. The findings enhance our understanding of the interaction between maternal and paternal psychological characteristics in contributing to children’s anxiety and depressive symptoms.

## Introduction

### The Role of Maternal and Paternal Depressive Symptoms and Emotion Dysregulation in Children’s Internalizing Problems

The prevalence of internalizing behavioral problems is notable as early as preschool years (Davis et al., 2015), where the rate ranges from 10% to 15% (Briggs-Gowan et al., 2004). Early childhood internalizing problems, including depressive and anxiety symptoms, do not represent a transient syndrome, but rather exhibit a tendency to become chronic and/or recurring problems (Bittner et al., 2007). Early internalizing symptoms not only hinder children’s daily functioning (Egger & Angold, 2006), but are also associated with long-term mental health challenges in adolescence and adulthood, such as anxiety and depressive disorders and increased risk of developing other psychiatric conditions later in life (Lee et al., 2022). Specifically, children’s internalizing symptoms and a family history of depressive disorders were the most reliable and significant predictors of depression later on, after controlling for demographic factors, comorbid disorders, and other risk factors (Luby et al., 2009). Hence, early identification of internalizing problems in preschool years is critical for timely interventions.

Research has extensively documented the contribution of maternal depression and emotion dysregulation to child internalizing symptoms, which encompass a range of emotional and behavioral difficulties such as anxiety and mood disorders (e.g. Bailer et al., 2017). Although early work emphasized a stronger association between maternal depression and child depressive symptoms compared to paternal influences (Connell & Goodman, 2002), recent studies highlight that fathers’ psychological health is just as critical to children’s social and emotional development (Gerhardt et al., 2020; Wilson & Durbin, 2010). Despite these findings, the paternal role in the context of parental depression and emotion dysregulation has been historically underrepresented in child psychopathology research (Goodman et al., 2011). While mothers’ psychopathology has been widely acknowledged as closely linked to children’s internalizing problems, family systems theory proposes that the emotional well-being of each family member, especially fathers, is interconnected, with the emotional health of any one individual potentially impacting the entire family dynamic (Bowen, 1978). Nonetheless, there remains a gap in understanding how fathers’ psychological factors might moderate the effects of maternal emotional challenges on the development of internalizing problems in children. Our study aims to fill this gap by exploring the nuanced interactions between paternal and maternal psychological factors and their collective influence on children’s internalizing problems.

### Maternal Depression, Maternal Emotion Dysregulation, and Children’s Internalizing Problems

Depression is characterized by persistent feelings of sadness and a lack of interest in previously enjoyable activities, affecting an individual’s overall functioning. On the other hand, emotion dysregulation refers to difficulties in managing emotional experiences or expressions in a way that is conducive to achieving one’s goals (Beauchaine & Gatzke-Kopp, 2012). While depression represents a clinical outcome often characterized by prolonged difficulties in downregulating negative emotion, such as sadness, and in upregulating positive emotion, emotion dysregulation refers to challenges in managing a broader array of emotional states.

As a transdiagnostic marker, emotion dysregulation distinguishes itself from depression by signaling regulatory challenges across a spectrum of psychopathologies (Beauchaine, 2015). Although emotion dysregulation can be an antecedent to depression, it is important to note that not all instances of dysregulated emotions culminate in clinical depression. Persistent emotion dysregulation, on its own, can prolong distress and may precipitate diagnosable mental health conditions, including but not limited to major depressive disorder, borderline personality disorder, bipolar disorder and generalized anxiety disorder (See reviews in Aldao et al., 2010).

Maternal depression and emotion dysregulation are interrelated factors that significantly affect a child’s risk for internalizing problems such as anxiety and depression. Depression can compromise a mother’s ability to provide emotional support, leading to her child’s increased risk of internalizing symptoms (Goodman et al., 2011). Furthermore, emotion dysregulation can disrupt maternal parenting practices, affecting emotional communication and responsiveness (Sanders et al., 2015). Moreover, Rutherford et al. (2015) have shown that a mother’s ability to regulate emotions, which is critically important for children’s social and emotional development, is often impaired by depression. In summary, maternal depression and emotion dysregulation, while conceptually distinct, are interrelated factors that both contribute to the risk of internalizing problems in children, albeit through different pathways.

Research indicates variability in the association between maternal depression and child internalizing problems, with a meta-analysis by Connell & Goodman (2002) suggesting that maternal depression only modestly affects the variance in these issues. This modest effect is also seen in the association between maternal emotion dysregulation and children’s internalizing issues (Zimmer-Gembeck et al., 2022).

Therefore, understanding the nuanced influences of both maternal and paternal emotion regulation is crucial in comprehensively addressing the multifaceted nature of children’s internalizing problems.

### The Moderating Role of Paternal Depression and Emotion Dysregulation

The interplay between paternal depression and maternal depression/emotion dysregulation is a crucial factor to consider when examining the development of internalizing problems in offspring. Goodman and Gotlib (1999) have highlighted various pathways through which fathers can influence the emotional development of children, particularly in the context of maternal depression. For instance, the presence of paternal depression may amplify the already detrimental effects of maternal depression, with studies showing more severe emotional difficulties in children when both parents are depressed, regardless of controlling for demographic variables (Paulson et al., 2006). Conversely, fathers who exhibit fewer depressive symptoms and engage in supportive parenting can serve as a protective ‘buffer’ against the negative effects of maternal emotional challenges. This suggests a dual role for fathers, where they can either contribute to a heightened risk of internalizing problems in children when experiencing high levels of depressive symptoms or provide resilience within the family system through positive engagement and emotional support, despite maternal emotional dysregulation (Goodman & Gotlib, 1999; Wilson & Durbin, 2010).

The moderating role of paternal emotion dysregulation in the association between maternal depression/emotion dysregulation and children’s internalizing problems is an area of research that warrants further exploration. While numerous studies have highlighted the pivotal role of paternal emotion dysregulation in shaping children’s developing of internalizing problems (e.g., Islamiah et al., 2023), there remains a noticeable gap in directly investigating how paternal dysregulation may interact with maternal depression or emotion dysregulation to impact children’s internalizing problems. This underexplored territory, however, gains credibility through insights from related constructs. For instance, Morris’s (2007) tripartite model underscores the importance of the family’s emotional climate, influenced by parental characteristics such as mental health and emotion regulation, in shaping children’s emotional development and susceptibility to problem behaviors (e.g., internalizing problems). Furthermore, research from meta-analyses shows that both parents’ emotion dysregulation and depressive symptoms are significantly associated with children’s internalizing symptoms (Goodman et al., 2011; Zimmer-Gembeck et al., 2022). In light of family system theory, which posits that families operate as intricate social systems with interdependent members, any disruption in one parent’s functioning, whether due to maternal depression/emotion dysregulation or paternal emotion dysregulation, can ripple through the entire family system (Bowen, 1978). Thus, exploring the unique and interactive contributions of both paternal depression/emotion dysregulation in the context of maternal depression and emotion dysregulation is essential for gaining a comprehensive understanding of the factors influencing children’s internalizing problems.

### Distinct Parental Influences on Childhood Depression and Anxiety

Internalizing problems in childhood, which could manifest as symptoms of anxiety and depression, are recognized as significant adjustment issues (Ollendick et al., 2008). Although anxiety and depression are interrelated–often co-occurring and arising from shared aspects of emotion dysregulation–they manifest differently. Anxiety is typically associated with excessive worry and fear, whereas depression is more often characterized by persistent sadness and anhedonia, or a diminished ability to experience pleasure in previously enjoyable activities (Campbell-Sills & Barlow, 2007). The high comorbidity among childhood disorders has led many researchers to utilize symptom checklists that aggregate internalizing disorders into broad categories, often failing to differentiate between the nuanced presentations of anxiety and depression (Hughes & Gullone, 2008). Historically, the impact of parental depression on childhood depression has been more extensively studied than its influence on childhood anxiety, despite both being internalizing problems (Goodman, 2007). Acknowledging this gap, it is essential to broaden research to explore how parental depression may contribute uniquely to childhood depression and anxiety, considering the subtle yet distinct parental influences on each condition.

Substantial research has highlighted the link between parental depression and offspring internalizing problems, such as anxiety and depression (e.g. Goodman et al., 2011). However, the nature of this association is complex and not uniformly consistent across different types of internalizing symptoms. For instance, studies have found a more pronounced connection between children’s depressive symptoms and elevated levels of parental psychopathology compared to children’s anxiety symptoms (Johnson & Greenberg, 2013). Besides, children of depressed mothers are more likely to display depressive over anxiety symptoms (Breslau et al., 1987). Conversely, other research suggests that parental depression might contribute nonspecifically to both depressive and anxiety symptoms in children, indicating a more generalized effect (See reviews in Goodman & Tully, 2006). The variability in these findings may be attributed to the potential role of child temperament characteristics, which has often been overlooked. Research indicates that individual differences in children’s negative emotionality, which is the tendency to show various forms of negative emotions, are more strongly associated with anxiety than with depressive symptoms (Jordan, 2012). This suggests that a child’s dispositional temperament may influence their susceptibility to depression or anxiety in the context of parental psychopathology. In addition, the importance of examining both maternal and paternal depression is underscored by their potential differential impacts on children. It is critical to consider the unique contributions of each parent’s depressive state, as they may affect child outcomes in distinct ways. For example, while maternal depression might influence child behaviors through the quality of maternal parenting, paternal depression may have an indirect effect on both internalizing and externalizing behaviors in children (Shafer et al, 2017). Recognizing these distinct pathways highlights the complex interplay of parental psychopathology and underscores the necessity of including both maternal and paternal perspectives to fully understand the influence on child internalizing symptoms.

### The Current Study

The present study extended prior research on the prospective association between maternal depression/emotion dysregulation and internalizing problems in children by investigating the moderating effects of paternal depressive symptoms and emotion dysregulation. Incorporating both maternal and paternal depressive symptoms and emotion dysregulation in the same model allowed for a simultaneous assessment of their individual and interactive effects. Moreover, this study utilized longitudinal data, allowing us to establish the directionality of the association and providing insights into the developmental changes of children’s internalizing problems. Additionally, instead of examining the broadband internalizing symptoms, this study examined maternal and paternal risk factors of depressive and anxiety symptoms in children separately, while also considering child temperamental emotionality.

**Figure 1:**
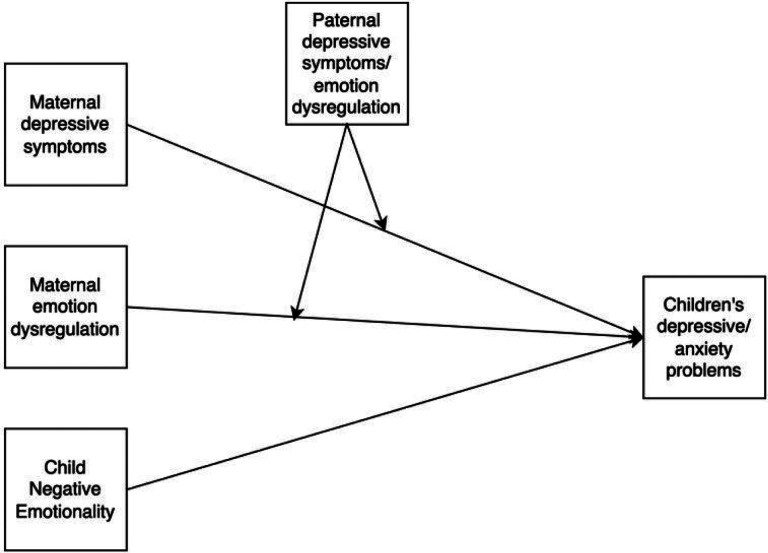
Conceptual model of the study

In this study, we examined the potential moderating role of paternal depressive symptoms on the connection between maternal depressive symptoms/emotion dysregulation and children’s subsequent development of depressive and anxiety symptoms. Building on this, we also investigated whether paternal emotion dysregulation might differently influence this relation, given that emotion dysregulation encompasses a broader spectrum of emotional problems while depressive symptoms is a more specific and clinically relevant measure that may have more direct relevance to children’s psychopathology. Our hypothesized model (Figure 1) delineates the roles of paternal depressive symptoms and emotion dysregulation, proposing that each may have a distinct moderating effect on the relation between maternal depressive symptoms/emotion dysregulation and child internalizing problems. We hypothesize that the link between maternal depressive symptoms/emotion dysregulation and the emergence of childhood depressive or anxiety symptoms would be amplified in the presence of heightened paternal depressive symptoms and/or emotion dysregulation, and attenuated when fathers display lower levels of these symptoms.

## Methods

### Participants

The participants of this study were drawn from a larger longitudinal study conducted in a Midwest city in the U.S. examining the effect of maternal depression on children’s autobiographic memory and emotion regulation. One hundred and twenty-five families participated in the study at the first time point (T1), when children were 4 years of age. Inclusion criteria for the mother included: 1) age 21 years or older; 2) having a biological child aged 3.5–4.0; 3) having no psychotic symptoms, a history of bipolar disorder, or substance use disorder within the past 6 months. Inclusion criteria for children included the absence of any developmental disorder or delay and an IQ score above 70. The second assessment (T2) occurred on average 14.29 months following T1.^[1]^

The sample of the current study included parents from 94 families (94 mothers and 91 fathers) that provided maternal or paternal reports on children’s internalizing problems at T2. Thirty-two families (24.8%) that participated in T1 did not return at T2. Half of the mothers (n = 47) had MDD during the child’s lifetime at T1. Children (46 girls) were 4.03 years old (SD = 0.17) at T1. Mothers were 35.18 years of age (*SD* = 4.50) and fathers, 37.61 (*SD* = 6.07) at T1. The majority of mothers (86.4%) and fathers (85.9%) identified as White, 9.1% of the mothers and 9.4% of the fathers identified as Black, while the rest identified as Native Hawaiian (1.1% mothers), American Indian (1.2% fathers), or mixed races (3.4% mothers and 3.5% fathers). The majority of the mothers (81.8%) and fathers (69.4%) had attained a college degree, of which 39.8% of the mothers and 20.5% of the fathers had a graduate or professional degree. The average income-to-needs ratio (household income divided by 100% of the federal poverty line for the number of individuals living in the house) was 3.58 (*SD* =1.66), meaning that the average family income was 3.58 times the federal poverty line, which could be an indicator of middle-class families (Conger et al., 1997). Families lost to attrition at T2 did not differ on any study variables or demographic variables at T1, including race, marital status, family income and child gender.

### Measures

#### Maternal/paternal depressive symptoms.

Depressive symptoms were measured by mother/father report on Beck Depression Inventory-2nd Edition (BDI; Beck et al., 1996) at T1. BDI is a 21-item, self-report questionnaire designed to evaluate the severity of depressive symptoms experienced within the past two weeks. It uses a scale ranging from 0 to 3, with higher scores indicating more severe depressive symptoms. It prompts self-reflection with statements such as “I do not feel sad” scored as 0, indicating no sadness, to “I am so sad and unhappy that I can’t stand it,” scored as 3, reflecting severe sadness. A sum score of depressive symptoms was then generated and included as a covariate in the analyses. The questionnaire has a high internal consistency (Cronbach’s alpha: Mother = .88; Father = .87).

#### Maternal/paternal emotion dysregulation.

Maternal and paternal emotion dysregulation was measured by the subscale, Limited Access to Emotion Regulation Strategies, of Difficulties in Emotion Regulation Scale (DERS; Gratz & Roemer, 2004) at T1. This subscale consists of 8 items (e.g., “When I’m upset, I believe I will end up feeling very depressed.”), rated on a 5-point Likert-type scale ranging from 1 (almost never) to 5 (almost always). Higher scores on this subscale indicate higher levels of emotion dysregulation. This subscale had high internal consistency (Cronbach’s alpha: Mother = .90; Father = .90).

#### Child depressive symptoms.

Children’s depressive symptoms were assessed by maternal and paternal report on the DSM-Oriented Affective Problems scale of the Child Behavior Checklist (CBCL; Achenbach & Rescorla, 2001) at both T1 and T2. The Affective Problems scale was developed with the purpose of identifying the existence of significant depressive symptoms (Achenbach et al., 2003). The 10-items are rated on a 3-point Likert-type scale ranging from 0 (not true) to 2 (very true), with higher scores indicating greater degrees of depressive symptoms. Example items include “cries a lot” and “looks unhappy without good reason”. The original scale had relatively low reliability only in father ratings at T1 (α = 0.59). One item (i.e., Overreacting) was deleted to improve scale reliability. After removing this item, Cronbach’s alphas for maternal report were .63 (T1) and .61 (T2) and for paternal report were .64 (T1) and 0.67 (T2). Maternal and paternal report on the same measure were moderately to highly correlated at both time points (T1: *r* = .40, *p* < .001; T2: *r* = .62, *p* < .001). Hence, a composite score was created by averaging the maternal and paternal report of children’s depressive problems, in order to generate a representative measure of the children’s depressive symptoms at both time points.

#### Child anxiety symptoms.

Children’s anxiety symptoms were assessed by maternal and paternal report on the DSM-Oriented Anxiety Problems scale of the Child Behavior Checklist (CBCL; Achenbach & Rescorla, 2001) at both T1 and T2. Ten items were included, using a 3-point Likert-type response scale ranging from 0 (not true) to 2 (very true). Higher scores are indicators of greater degrees of anxiety problems. Example items include “does not want to sleep alone” and “nervous, highstrung, or tense”. This scale demonstrated acceptable internal reliability at both time points (Cronbach’s alpha: Mothers: T1 = .69; T2 = .77; Fathers: T1 = .70; T2 = .68). Similarly, at both time points, a representative measure of the children’s anxiety symptoms was obtained by creating a composite score, averaging maternal and paternal reports, given that there was a strong positive correlation between maternal and paternal reports at both time points (T1: *r* = .61, *p* < .001; T2: *r* = .54, *p* < .001).

#### Child Negative Emotionality.

Child Negative Emotionality was assessed at Time 1 using the Negative Emotionality scales of the Children’s Behavioral Questionnaire (CBQ; Rothbart et al., 2001), as reported by both mothers and fathers. The scale comprised 36 items rated on a 7-point scale, with 1 being ‘extremely untrue’ and 7 ‘extremely true,’ and included subscales for anger/frustration, discomfort, fear, sadness, and soothability (reversed). Examples of items are ‘Gets angry when told s/he has to go to bed’ and ‘Becomes upset when loved relatives or friends are preparing to leave after a visit.’ The scale showed good internal consistency for both parent reports (Cronbach’s alpha: Mothers: .88; Fathers: .88). A composite score of child negative emotionality was created by averaging the mother and father reports, which were strongly positively correlated (*r* = .63, *p* < .001).

#### Income-to-needs Ratio.

The family income was initially reported on a scale of 1 to 12 by mothers at T1, and an income-to-needs ratio was computed by dividing the household income by the federal poverty line for the household size during the study year.

### Data Analysis

Path analyses were performed using Mplus, Version 8.2 (Muthen & Muthen, 1998–2017). Observed variables include maternal and paternal depressive symptoms at T1, maternal and paternal emotion dysregulation at T1, child negative emotionality, income-to-needs ratio and children’s depressive/anxiety problems at T1 and T2. Due to maternal history of MDD being an inclusion criterion for the larger study, and approximately half of the mothers having a history of MDD in this study, its effect was initially examined in the preliminary analysis. However, due to its high correlation with maternal depressive symptoms (*r* = .58, *p* < .001) and its lack of additional explanatory power beyond maternal depressive symptoms, it was subsequently excluded from the final analysis.

Since the T2 measures served as the dependent variable, families with T1 but not T2 cannot be imputed/estimated for analysis. Including imputed values for the dependent variables in the analysis would only introduce unnecessary distortion to these estimates (Von Hippel, 2007). Therefore, only 94 mothers and 91 fathers that provided reports on children’s internalizing problems at T2 were included in the analyses. Data that were missing on father report (n = 3, 3.2%) were addressed using Maximum Likelihood estimate. Given the modest sample size and the increased complexity associated with including multiple outcome variables in the same model, it is justifiable to conduct separate analyses for each of the four models to examine the moderating role of paternal depressive symptoms/emotion dysregulation on children’s depressive and anxiety symptoms (Memon et al., 2019). This approach helps to prevent the issue of overfitting, where the model becomes too specifically tailored to this particular dataset, limiting its applicability elsewhere (Fan et al., 1999). The models tested the moderating role of paternal depressive symptoms and emotion dysregulation on the relation between maternal depressive symptoms/emotion dysregulation and later depressive and anxiety symptoms in children, also taking into account the effects of child negative emotionality. Income-to-needs ratio was included as a covariate, as it was typically associated with maternal depression and child emotional outcomes (Goodman et al., 2011). Children’s depressive or anxiety symptoms at T1 were controlled for as covariates in the models. The interaction effects were further probed by testing and plotting the slopes of the association between the predictor and outcome variables at mean, low (−1SD), and high (+1SD) levels of the moderator variable).

## Results

Descriptive statistics and the bivariate correlations of the study variables are shown in Table 1. Maternal depressive symptoms and emotion dysregulation were positively correlated, and so were paternal depressive symptoms and emotion dysregulation. However, maternal depressive symptoms or emotion dysregulation did not significantly correlate with the paternal depressive symptoms or emotion dysregulation, and vice versa. Maternal depressive symptoms at T1 were positively correlated with children’s anxiety problems at both time points, but with children’s depressive symptoms only at T1. Maternal emotion dysregulation at T1 was positively correlated with children’s concurrent depressive and anxiety symptoms, but not with either depressive or anxiety symptoms later. As for paternal emotion dysregulation and depressive symptoms, they both correlated positively with children’s depressive problems but not anxiety problems at T1.

Two separate path models were estimated to examine the moderating effects of paternal depressive symptoms and paternal emotion dysregulation on each type of internalizing problem. Results are presented in Tables 2 and 3. Due to all models being saturated, with every possible relations between variables estimated, fit indices were perfect and thus omitted from the report.

**Figure 2:**
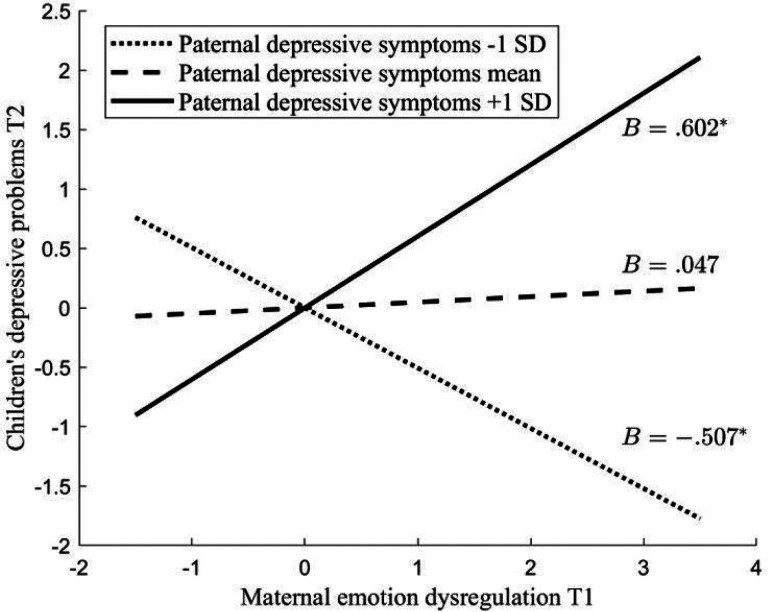
Interaction between maternal emotion dysregulation and paternal depressive symptoms

We first tested the moderating role of paternal depressive symptoms in the association between maternal depression/emotion dysregulation and children’s depressive and anxiety problems (Table 2). In the model using children’s depressive problems as the outcome variable, 44.3% of the variance in children’s depressive problems was explained by this model. No significant main effects were found for maternal or paternal depressive symptoms and maternal emotion dysregulation, except for the children’s earlier depressive symptoms. A significant interaction emerged between paternal depressive symptoms and maternal emotion dysregulation (*B* = 0.554, *SE* = 0.202, *p* = .006). The interaction effect was further explored by examining the associations between maternal emotion dysregulation and child depressive symptoms at high (+1SD), mean, and low (−1SD) levels of paternal depressive symptoms (Fig. 2). When fathers had high levels of depressive symptoms (1SD above the mean), there was a positive association between maternal emotion dysregulation and children’s later depressive problems (*B* = 0.602, *SE* = 0.277, *p* = .030). When fathers had low levels of depressive symptoms (1SD below the mean), there was a negative association between maternal emotion dysregulation and children’s later depressive problems (*B* = − 0.507, *SE* = 0.246, *p* = .039). Notably, this association was not significant for fathers with medium levels of depressive symptoms. In the model using children’s anxiety problems as the outcome variable, 50.5% of the variability observed in children’s anxiety problems can be explained by this model. In addition to children’s anxiety problems at the previous time point, child negative emotionality also showed significant main effect in this model. No other significant main effect or interaction effect was found.

Next, the moderating role of paternal emotion dysregulation was evaluated (Table 3). Here, the models explained 40% and 49.3% of the variance in children’s depressive and anxiety problems, respectively. Apart from the prior time point symptoms, no significant main or interaction effects were detected.

## Discussion

The goal of the current study was to investigate the moderating role of paternal depressive symptoms/emotion dysregulation in the relations between maternal depression/emotion dysregulation and children’s later internalizing problems using a longitudinal approach. We found that paternal depressive symptoms significantly moderated the association between maternal emotion dysregulation and children’s later depressive symptoms. However, paternal emotion dysregulation did not exert moderating effects on the connections between maternal depressive symptoms/emotion dysregulation and children’s subsequent depressive or anxiety symptoms. These findings underscore the complexity of the relationships between nuanced parental emotional factors and children’s internalizing problems and highlight the need to consider the specific parental emotional factors and children’s emotional outcomes when examining the impact of maternal emotional well-being.

Above all, our findings suggested the moderating role of paternal depressive symptoms in the relation between maternal emotion dysregulation and children’s depressive problems. Specifically, when fathers had high levels of depressive symptoms, maternal emotion dysregulation was linked to high levels of depressive problems in children one year later. This aligns with prior research, which underscores that fathers with elevated depressive symptoms contribute to a less favorable child-rearing environment, heightening the risk of emotional problems in their children, especially when mothers face challenges in managing their emotions and do not provide the nurturing qualities known to support healthy emotional development (Goodman et al., 2011). Also consistent with the hypothesis, when fathers had average levels of depressive symptoms, the positive association between maternal dysregulated emotion and children’s subsequent depressive symptoms disappeared. This finding suggests that fathers’ psychological health plays a pivotal role, especially when maternal emotional regulation is compromised. The emotional stability and well-being of fathers may act as a critical counterbalance, potentially dampening the impact of maternal difficulties on the child’s development. Such paternal support could be integral in offering children consistent, positive interactions and emotional engagement, which are essential when maternal emotional support is less available (Goodman et al., 2011). The significance of a father’s emotional health extends beyond its inherent value, serving as a potential mitigating factor against the challenges posed by a mother’s emotional dysregulation, and thus playing a protective role in a child’s developmental trajectory. Unexpectedly, however, our results indicated that when fathers had low levels of depressive symptoms, mothers with higher emotion dysregulation had children with lower depressive problems one year later. It highlights the complexity of family dynamics and suggests that when fathers demonstrate lower depressive symptoms, their positive psychological state may not only offset the negative influence of maternal emotion dysregulation but could also actively contribute to a more resilient environment. The presence of at least one emotionally stable parent may help children develop effective coping mechanisms and resilience, which could reduce the occurrence of depressive problems (Fitzgerald et al., 2021). Essentially, a father with good mental health might compensate for a mother’s emotion dysregulation by providing additional emotional security, modeling effective emotion regulation strategies, and fostering a supportive atmosphere that helps children navigate and adapt to their mother’s emotional challenges (Goodman et al., 2011; Martin et al., 2022). This supportive environment could encourage children to develop independent emotional regulation skills, leading to lower rates of depressive problems over time (Islamiah et al., 2023). Nonetheless, the nuanced dynamics of this protective effect, particularly in the context of significant maternal emotion dysregulation, warrant further investigation to delineate under which circumstances paternal depressive symptoms may safeguard against the intergenerational transmission of emotional difficulties.

The hypothesis that paternal depression would moderate the association between maternal depression and children’s internalizing problems was not substantiated. Although past studies found paternal and maternal depression interacted in predicting child internalizing problems (e.g., Goodman et al, 2007), the current study did not find similar moderating effects of paternal depressive symptoms using longitudinal data and both parents’ reports of children’s emotional problems. As we included both maternal depressive symptoms and emotion dysregulation, which were highly correlated, it is possible that the interaction between paternal and maternal depressive symptoms did not explain much variance in child depressive symptoms beyond what was already accounted for by the interaction between paternal depressive symptoms and maternal emotion dysregulation. However, in the model with child anxiety symptoms as the outcome variable, we found a direct effect of child negative emotionality, which involves a heightened response to fear and distress, which aligns more with anxiety and may render children more susceptible to such symptoms in response to direct environmental triggers rather than the broader emotional context of the home (Nigg et al., 2006). This sensitivity could mean that for children predisposed to high negative emotionality, parental emotional states may play a less significant role in anxiety development compared to other more immediate factors, such as direct stressors or specific traumatic events (Lonigan et al., 2001). Thus, these nuances underscore the complexity of parental influence and highlight the importance of considering individual differences in child temperament when examining the transmission of anxiety and depressive symptoms.

The moderating effect of paternal emotion dysregulation in the relation between maternal depression, maternal emotion dysregulation, and children’s depressive and anxiety problems, were not found. While previous research posited that paternal emotion dysregulation could significantly influence the development of children’s internalizing problems (Islamiah et al., 2023), our study did not demonstrate significant main effects from either parent on children’s depressive or anxiety symptoms. This could suggest a more complex interplay between maternal and paternal emotional regulation than previously understood, with other uninvestigated factors potentially influencing these dynamics. These findings parallel previous meta-analytic results indicating a modest effect size for the association between maternal depression and child internalizing problems (Connell & Goodman, 2002), as well as reported heterogeneity in effect sizes in the research linking parental emotion dysregulation with child adjustment (Zimmer-Gembeck et al., 2022). Additionally, factors that could potentially moderate the effects of maternal depression on children’s later internalizing problems were not explored in this study. For instance, the duration, progression, and onset of maternal depression are known to significantly affect child outcomes (Goodman & Gotlib, 1999), and the correlation between maternal and child depressive symptoms is posited to be more pronounced in clinical samples (Goodman et al., 2011).

The limitations of the current study indicate areas for future research to explore. First, the generalizability is limited in that half of the mothers had a history of MDD due to the sampling strategy of the larger study, and the participants were primarily White, well-educated parents from middle-class families. The relative homogeneous sample, coupled with a modest sample size, might have restricted the variance in the measures and prevented us from identifying potential direct and indirect associations of maternal/paternal depressive symptoms and dysregulated emotion with children’s internalizing symptoms. Future studies with larger and more diverse samples are needed to further elucidate the complex dynamics underlying the associations examined in this study. Apart from that, it is important to note that maternal and paternal depression/emotion dysregulation and child temperamental characteristics and emotional outcomes, were solely assessed through self- and parent-report measures. Additionally, the depressive and anxiety symptoms have relatively low internal consistency, as measured by CBCL-DSM-oriented scale, which is a pattern noted in previous research studies (e.g., Soltani et al., 2023). This reliance on parental report limits the scope of available options and introduces the potential for response bias. Previous research has demonstrated that parental assessments of children’s behaviors can be affected by their own psychopathology (e.g., Gartstein et al., 2009) and parenting behaviors (e.g., Webster-Stratton & Hammond, 1988), potentially introducing bias. To minimize potential parent-report bias, we incorporated the responses of both mothers and fathers in our assessment of child temperamental characteristics and outcomes. However, to overcome these limitations, future research should consider employing diverse methods such as observations, which offers a more objective assessment of child characteristics. Lastly, the role of maternal emotion dysregulation as a potential mediator in the relation between maternal depression and child internalizing problems should be considered. Specifically, higher levels of maternal depression are linked to increase difficulties in accessing emotion regulation strategies, which, in turn, are associated with elevated levels of child’s internalizing problems (Ip et al., 2021). However, as our study only had two time points, it is not ideal to examine the mediating role of maternal emotion dysregulation.

Notwithstanding these limitations, this study possesses several noteworthy strengths. First, this study expands the literature that considers paternal emotional characteristics in examining the impact of the maternal risk factors on child outcomes. It also endeavored to identify the moderating role of paternal factors in the associations between maternal depressive symptoms/dysregulated emotion and children’s later outcomes. Second, by utilizing longitudinal data, this study enables the examination of the relation between maternal depression/emotion dysregulation, paternal depression/emotion dysregulation and child internalizing problems over time, capturing developmental changes of children’s internalizing problems and providing deeper insights into the impact of parents’ psychological well-being on their children. Third, a notable strength of this study is the inclusion of assessments on children’s depressive and anxiety problems from both maternal and paternal reports. As previous studies indicated that anxiety and depressive symptoms are best represented by separate though correlated constructs (e.g. Lonigan et al., 2003), our methodology harness multiple perspective to gain a more comprehensive understanding of children’s emotional well-being and can account for potential biases or discrepancies that may arise from relying on a single source of information. This approach enhances the validity and reliability of our findings and provides a more nuanced picture of children’s emotional functioning within the family context. Additionally, building on previous research that has typically focused on assessing either depressive or anxiety symptoms in children, or a combination thereof, this study separately examined the role of parental emotional regulation challenges in each type of symptoms in young children. Our findings contribute to a growing body of evidence suggesting that depression and anxiety represent related but distinct constructs in early childhood (e.g. Johnson & Greenberg, 2013). Furthermore, our results indicate that parental emotional regulation challenges can have varying impacts on young children’s experiences of depression and anxiety, underscoring the need to consider these emotional disorders separately when evaluating family dynamics and child outcomes.

In conclusion, this study uncovered a nuanced interplay between maternal emotion dysregulation and paternal depressive symptoms in shaping children’s internalizing problems. This intricate dynamic highlights the complexity of parental influences on children’s well-being. Recognizing the multifaceted interplay among these variables provides a foundation for future research and interventions to offer more informed and effective support for children and families in fostering positive developmental outcomes.

## Data Availability

The data and materials are not available for the public.

## Footnotes

1. The interval between the two time points was longer than the intended 9–12 months due to the impact of the COVID-19 pandemic.
